# Response Inhibition Is Facilitated by a Change to Red Over Green in the Stop Signal Paradigm

**DOI:** 10.3389/fnhum.2016.00655

**Published:** 2017-01-04

**Authors:** Shawn Blizzard, Adriela Fierro-Rojas, Mazyar Fallah

**Affiliations:** ^1^Visual Attention and Perception Laboratory, School of Kinesiology and Health Science, York UniversityToronto, ON, Canada; ^2^Centre for Vision Research, York UniversityToronto, ON, Canada; ^3^Department of Psychology, Benemérita Universidad Autónoma de PueblaPuebla, Mexico

**Keywords:** attention, visual perception, executive function, response inhibition, response execution, stop-signal task

## Abstract

Actions are informed by the complex interactions of response execution and inhibition networks. These networks integrate sensory information with internal states and behavioral goals to produce an appropriate action or to update an ongoing action. Recent investigations have shown that, behaviorally, attention is captured through a hierarchy of colors. These studies showed how the color hierarchy affected visual processing. To determine whether the color hierarchy can be extended to higher level executive functions such as response execution and inhibition, we conducted several experiments using the stop-signal task (SST). In the first experiment, we modified the classic paradigm so that the go signals could vary in task-irrelevant color, with an auditory stop signal. We found that the task-irrelevant color of the go signals did not differentially affect response times. In the second experiment we determined that making the color of the go signal relevant for response selection still did not affect reaction times(RTs) and, thus, execution. In the third experiment, we modified the paradigm so that the stop signal was a task relevant change in color of the go signal. The mean RT to the red stop signal was approximately 25 ms faster than to the green stop signal. In other words, red stop signals facilitated response inhibition more than green stop signals, however, there was no comparative facilitation of response execution. These findings suggest that response inhibition, but not execution, networks are sensitive to differences in color salience. They also suggest that the color hierarchy is based on attentional networks and not simply on early sensory processing.

## Introduction

Our actions are generated by integrating sensory information into the response execution and inhibition networks. This produces a new action appropriate to the environment or takes an action which is already being carried out and updates or inhibits it. The prefrontal cortex works with the basal ganglia to control response selection and suppression (Mink, 1996; Gondo et al., 2000; Nambu et al., 2002; Chao et al., 2009; Hege et al., 2014; Jahfari et al., 2015; Rae et al., 2015).

Given that vision is a hallmark of the human experience it is not surprising that visual signals are used to usher, change or stop a particular behavior. For example, a traffic light turning red is designed to capture the attention of a driver and will, hopefully, bring about a swift movement of the foot from the accelerator to the brake pedal. However, if a driver is approaching a red light and it turns green, movement towards the brake pedal is countermanded and the foot stays on the gas. There are many associations dependent on color. In general, red depicts danger, whereas green depicts safety. While there are certainly learned associations between color and response selection, there is evidence to suggest that the color of a visual signal alone could alter its effectiveness (Lindsey et al., 2010; Tchernikov and Fallah, 2010; Pomerleau et al., 2014).

### Color Hierarchy

Tchernikov and Fallah (2010) measured smooth pursuit eye movements after subjects made a saccade to two superimposed moving random-dot-kinematograms (*RDK*s*)* segregated using color. Smooth pursuit target selection depended on a color hierarchy of red, green, yellow and blue which describes the inherent priority (salience) we give to the different colors. The velocity of pursuit was dependent on the difference in salience between the two objects. Thus color intrinsically drives attentional capture and differences in intrinsic color salience drive differences in motor output. Further support comes from Lindsey et al. (2010) where it was found that target detection responses in a visual color search task were fastest for warmer (i.e., redder) colors than for cooler (i.e., bluer) colors.

Electrophysiological evidence has also been found to complement these behavioral findings. In a study of event-related potentials (*ERPs*), Pomerleau et al. (2014) found that the *N2PC* waveform appeared earliest for red stimuli (205 ms) compared to blue (223 ms), green (250 ms) and yellow stimuli (253 ms). The *N2PC* has been described as an indication of spatial filtering or surround suppression (Luck and Hillyard, 1994), of target feature enhancement at an attended location (Mazza et al., 2009), and as an index of the localization of a target prior to the deployment of attention (Tan and Wyble, 2015). They also found that the *PPC* (positive posterior contralateral) waveform had greater amplitude for red than for blue or green but the same amplitude when compared to yellow. Further comparisons of red and yellow showed that the larger amplitude was spread over a wider area for red when compared to yellow, thus corroborating the color hierarchy (Tchernikov and Fallah, 2010). Finally, the *PTC* (positive temporal component) waveform had greater positivity in response to red than for any other color. The *PTC*, located over the temporal lobes, is thought to be an indication of activity in the ventral visual stream. These results suggest that red stimuli elicit both an earlier and greater response and that, especially with regards to the *N2PC* waveform, the behavioral effects of red cited above may be due to red being preferentially processed.

When comparing the four main colors these studies show evidence for a color hierarchy of processing speed and strength. Behaviorally speaking, colors capture attention in a hierarchy of red, green, yellow and blue. The timing effects however were more ambiguous with red producing the earliest N2PC, followed by blue, and then green/yellow. It is possible that this difference may come from task demands. While in Lindsey et al. (2010) and Tchernikov and Fallah (2010) subjects were required to make eye movements to targets and color was a relevant part of the task, this was not the case in Pomerleau et al. (2014). Thus, color salience effects on processing may be dependent on task demands, where the object is selected as a whole or color is relevant to the response.

These findings have important implications for behavioral control because it suggests that executive functions, which are dependent on sensory input, may also be faster for red signals than for those of other colors. Specifically, the color hierarchy may drive differences in attentional allocation and, therefore, may influence higher level behavioral functions.

### Behavioral Control and the Stop Signal Paradigm

Motor execution and inhibition represent two particularly important facets of executive function as they allow for an efficient way of acting upon the environment while also ensuring that alternative but perhaps inefficient or otherwise inappropriate actions are suppressed. The interaction between execution and inhibition has typically been studied using what is known as the stop signal paradigm.

This task was used by Logan and Cowan (1984) as a way of synthesizing the vast amount of literature on both behavioral and cognitive control. In this task, participants are required to respond when presented with a go-signal but must countermand this response when presented with a stop-signal, e.g., pressing a button in response to a visual stimulus appearing and countermanding that response when an auditory tone was subsequently presented. Their findings provided support for what they called the “horse-race model” of behavioral control, also supported by more recent studies (Hanes et al., 1998; Kalanthroff et al., 2013; Gulberti et al., 2014). In this model, behavioral execution and inhibition are controlled by independent processes which compete to reach threshold. When one of the processes wins the race the other process is blocked from continuing. Response inhibition takes less time than response execution. So by varying the delay in presenting the stop signal after the go signal, the minimum amount of time needed for response inhibition (stop signal reaction time; *SSRT*) can be determined. Through the use of the stop-signal task (SST; in addition to the distinct but related go/no-go task), various studies have shown that inhibition relies upon a network of frontal regions (for example, the right inferior frontal gyrus, the middle frontal gyrus and the supplementary motor area with the motor cortex as a target of cortical inhibition) as well as the indirect and hyperdirect pathways of the basal ganglia (e.g., Nambu et al., 2000, 2002; Li et al., 2006; Aron et al., 2007; Swann et al., 2009, 2012, 2013; Cai et al., 2012; Mattia et al., 2012; Krämer et al., 2013; Jahfari et al., 2015; Fonken et al., 2016). Researchers have also found that inter- and intra-individual differences in the allocation of attention to sensory signals, as opposed to the success or failure of the fronto-basal inhibitory process, may provide an explanation for successful vs. unsuccessful response countermanding. Bekker et al. (2004) provided indirect evidence for this by comparing the ERPs evoked during successful and unsuccessful stop trials using auditory stop signals. Successful countermanding resulted in a positivity over frontal electrodes at 300 ms following the onset of the stop signal. Indeed, this positivity was greater for successful vs. unsuccessful stopping. Importantly for the attentional account of inhibitory control, there was a negative waveform over temporal electrodes at 100 ms that was greater for successful relative to unsuccessful countermanding. The authors note that this negativity has been argued to be a marker of selective attention (Hillyard et al., 1973).

This attentional explanation has garnered more recent direct support as well. In Verbruggen et al. (2014) a go signal comprised of two words, one for natural and one for non-natural objects, was presented centrally. To ensure that attention was allocated centrally, participants had to report whether the word described a natural or non-natural object. Stop signals were either presented centrally or as a frame located around the periphery of the display. In some trials, the go signal was accompanied by numerous letter-dyad distractors presented in random locations with these locations changing once every 100 ms to ensure a sustained cognitive load. As expected, estimated SSRTs were longer on distraction trials than on non-distraction trials. During trials with a distraction, estimated SSRTs were significantly longer when the stop signal was presented in the periphery relative to when it was presented centrally. This demonstrates that attention may be necessary for successful countermanding since the stop signal located outside of the locus of attention took significantly longer to process and, therefore, usher successful countermanding. These attentional effects would suggest that differences in salience across the color hierarchy may also drive effects in the SST.

Recent magnetoencephalographic (MEG) research has shown that success on the SST also depends on the quality of early sensory processing. Boehler et al. (2009) showed that a waveform negativity (N1) over occipito-temporal regions could be used to index eventual success or failure on the SST. Specifically, failures to countermand on stop trials were preceded by a greater N1 amplitude in response to the go signal relative to the N1 amplitude in response to the stop signal. The inverse was true on successful stop trials. This is important because it suggests that the amplitude of the response to a signal can affect the reaction time (RT) for a response to that signal. Furthermore, it suggests that responses to a red go signal may be faster than to a green go signal.

An important aspect of color beyond attentional salience that could have an effect in the SST is the learned association between color and particular actions from our built environment. For example, red traffic lights and caution signals (e.g., at railroad crossings) are used to signal that a driver should depress the brake pedal to slow-down and stop their vehicle. Green traffic lights, on the other hand, are used to signal that a driver should depress the gas pedal to accelerate or that they should continue driving through an intersection. In other words, red may often be associated with stopping while green may be associated with going. Attentional salience notwithstanding, given these associations, we might expect that red signals would provide for faster response inhibition while green signal would provide for faster response execution.

### Present Study

There are several differences in methodology to note between the present study and the previous color hierarchy studies. First, responses in Tchernikov and Fallah (2010) were reflexive eye movements made directly on the colored stimulus. In Lindsey et al. (2010), responses were made following overt search of a stimulus array. In these studies, it is assumed that the oculomotor system, which is tightly linked to the attentional control system (e.g., Moore and Fallah, 2001), plays a role in the speeded target selection and search. A similar mechanism could have been at play in Pomerleau et al. (2014), where participants needed to move their eyes about an array in order to count target stimuli. If, however, the color hierarchy is primarily driven by bottom-up visual processing and does not rely on attention and the oculomotor control system specifically we would expect to see an effect of color on performance in any task, including the SST.

For the present study, we predict that the attentional effects of color should have an effect specific to the executive function tied to that stimulus. Thus, colored go-signals should affect response execution, but not inhibition, whereas colored stop-signals should affect response inhibition, but not execution. This is because the two processes operate in parallel in the horse-race model that describes performance in the SST. In other words, consistent with the color hierarchy subjects should react more rapidly to red than other colors and this should either facilitate or impede response countermanding depending on the role of the red stimulus. In addition, we hypothesize that the attentional effects of color should affect go signal response accuracy when the go signal is manipulated by task relevant color. Specifically, participants may be more accurate for red go signals relative to green when responses are made according to color.

## Materials and Methods

### Participants

All participants had either normal or corrected-to-normal vision and successfully passed Ishihara’s Test for red-green color blindness (Ishihara, 2006). Twenty-four student volunteers from York University completed Experiment 1 (14 females, 10 males; ages 20–41). Three participants were excluded from data analysis because their response accuracy was below 50% on go trials (see Table 1). Thirty students from an introductory psychology course at York University completed Experiment 2 (18 females, 12 males; ages 18–23). Participants in Experiment 2 received partial course credit for their participation. Eight of these participants were excluded from data analysis because their response accuracy was below 50% on go trials (Table 1). As such, the data from 22 participants (14 females, 8 males; ages 18–23) were analyzed. It is important to note that the number of participants who met the analysis exclusion criteria may have been higher in Experiment 2 due to the increased number of possible go signal responses. Twenty-two participants (21 of whom completed Experiment 1) completed Experiment 3. Three of these participants were excluded from data analysis because their response accuracy was below 50% (Table 1). The final analyses for Experiments 1 and 3 included the data from the 19 participants that successfully completed both Experiments (9 females, 10 males; ages 20–41). In accordance with the Declaration of Helsinki all participants gave written informed consent prior to participation. All experiments were approved by York University’s Human Participants Review Committee.

**Table 1:** **Mean and standard deviations by color for aggregate go signal accuracy rates based upon overall accuracy rates**.

Experiment	Go signal color	>50%	<50%
**Experiment 1**	White	92.68% (4.94)	45.32% (4.35)
	Red	92.53% (4.73)	43.24% (6.24)
	Green	94.16% (4.17)	44.89% (6.19)
**Experiment 2**	White	80.59% (11.04)	29.68% (3.91)
	Red	82.64% (6.87)	31.12% (2.26)
	Green	82.18% (6.18)	29.96% (2.19)
**Experiment 3**	White	93.64% (4.65)	46.91% (3.14)

### Equipment

Participants sat 57 cm from an 18” CRT monitor (Dell M991, refresh rate = 60 Hz, resolution = 1280 × 1024) with their head stabilized by a headrest (UHCO Tech). Experimental control was maintained by Presentation (Neurobehavioral Systems). For Experiments 1 and 3, responses were made using left and right arrow keys on a keyboard. For Experiment 2, responses were made using a serial response box comprised of colored buttons (RB-540 serial response box, Cedrus Corporation).

### Stimuli and Procedure

#### Experiment 1

In this experiment we tested whether task irrelevant color would affect response times. Specifically, we tested whether a red go-signal would improve response times when compared to a green or white go-signal. The go-signals were isoluminant red (CIE *X* = 46.8, *Y* = 24.52, *Z* = 2.75), green (CIE *X* = 12.02, *Y* = 24.42, *Z* = 4.42) and white (CIE *X* = 23.11, *Y* = 24.30, *Z* = 33.74) arrows. Isoluminance was determined physically using a spectrophotometer. The stop-signal was an auditory tone (72 dB, duration = 916 ms).

**Figure 1:**
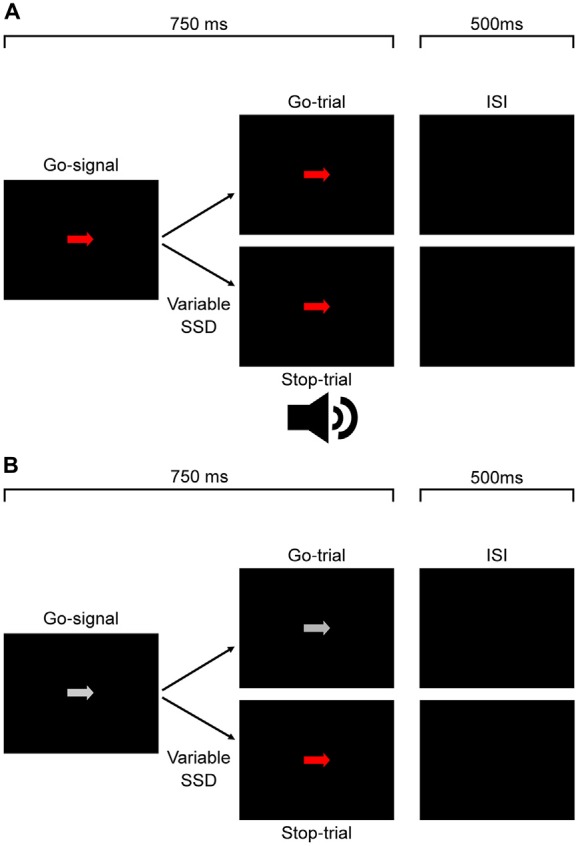
**Experimental protocol for Experiments 1, 2 (A)** and 3 **(B).** For Experiment 1 **(A)**, participants responded to the direction of a white, red (depicted) or green go signal arrow. On a subset of trials this was followed after a variable delay by an auditory stop signal which signaled participants to countermand their response. The experiment protocol was the same for Experiment 2 **(A)**, except participants responded to the color of the signal and not the arrow direction. In Experiment 3 **(B)**, participants responded to the direction of a white go signal arrow. On a subset of trials this was followed after a variable delay by a color change from white to either red or green (equal proportions, pseudorandomly interleaved) which signaled participants to countermand their response.

Figure 1A shows the time course of both stop- and go-trials in Experiment 1. On all trials, an arrow randomly pointing either right or left was displayed at the center of the monitor. The arrow was pseudorandomly chosen to be isoluminant red, green or white. Participants were required to respond as fast as possible using the corresponding arrow key (go-trial). On a subset of trials (stop-trials) the arrow was followed by the auditory stop-signal and participants were required to withhold their response. Participants received visual feedback for errors on arrow discrimination, responses on stop-trials and failures to respond within the 750 ms time window on go-trials.

The delay between the go- and stop-signals (stop-signal delay, SSD) began at 50 ms for each color and then varied using a staircase design. Each block consisted of 6 go-trials and 3 stop-trials for each color totaling in 27 trials per block. Trial type and go-signal color were pseudorandomly interleaved within each block. Each time a participant was successful in countermanding their response on a stop-trial the SSD for that color condition would increase, giving them less time for response inhibition on subsequent stop-trials. If they failed to countermand their response, the SSD would decrease, giving them more time for response inhibition on subsequent stop-trials. The step size of the SSD change started at 50 ms for the first stage of the staircase. When performance on a stage reached a double reversal, the step size decreased for the next stage (20 ms, 10 ms and 5 ms). The experiment ended after all stages were completed or when 102 stop-trials (34 blocks) were completed for each go-signal color.

#### Experiment 2

In this experiment we tested whether task relevant color would affect response times. The stimuli and procedure were the same as in experiment one except instead of responding according to the direction of the go-signal arrow participants were now required to respond with the button that corresponds to the color of the arrow. They were instructed to ignore the direction of the arrow. Figure 1A shows the time course of both go- and stop-trials for Experiment 2.

#### Experiment 3

In this experiment, we tested whether the color salience of a visual stop-signal would affect response inhibition, changing the time needed to countermand the response. The procedure was the same as Experiment 1 except for the following modifications. The go-signal arrows were always white and the auditory stop signal was replaced by an isoluminant color change of the white arrow to either red or green. Each block consisted of six go-trials and three stop-trials for each stop color condition totaling in 18 trials per block. Trial type and go-signal color were pseudorandomly interleaved within each block and the SSD for each color varied according to the same staircase procedure as in Experiment 1. It is important to note that since the stop signal was a color change, the true SSD was based on monitor refresh rate. As such, the SSDs were recalculated based on time of each refresh cycle. These recalculated SSD values were used in all analyses for Experiment 3. Figure 1B shows the time course of both stop- and go-trials in Experiment 3.

### Data Analysis

#### Experiments 1 and 2

The first block for each color was a practice block and the data was excluded from analysis. Response times which fell outside of ±2.5 standard deviations were removed from further analysis. Mean RTs were calculated as the average response time on go-trials for each go-signal color. Individual coefficients of variance (ICOVs) were calculated separately for each go-signal color as the standard deviation of response times divided by the mean response times for that participant. SSRTs were calculated using the integration method outlined by Logan and Cowan (1984). Specifically, SSRTs were calculated by finding the *n*th RT from a participant’s go signal RT distribution where the *n*th RT was determined by the number of RTs in the distribution multiplied by the proportion of correctly withheld responses during stop trials. The participants overall mean SSD was then subtracted from the *n*th go signal RT to produce the SSRT value. Response accuracy was calculated as the proportion of trials in which participants responded using the correct go signal response button. Repeated measures ANOVAs, with go-signal color as the independent variable, were conducted separately for mean response times (RT), mean ICOVs, mean SSD, SSRT and go signal response accuracy. Paired *t*-tests were also used to test the planned comparisons between red and green for each measure.

#### Experiment 3

A repeated measures paired *t*-test with stop-signal color as the independent variable was conducted for SSRTs. SSRTs were calculated using the same methods as described above for Experiments 1 and 2. Because there was only one go-signal color, mean RTs and ICOVs were not submitted to statistical testing.

## Results

Table 1 shows the mean aggregate accuracy of go responses (i.e., errors constituted go signal response omissions or incorrect go signal button response) for Experiments 1, 2 and 3 according to whether or not participants were excluded due to high error rates. Table 2 shows the mean SSD according to go signal color in Experiments 1 and 2 and according to stop signal color in Experiment 3.

**Table 2:** **Mean and standard deviation for stop-signal delays (SSDs) according to go signal color in Experiments 1 and 2 and according to stop signal color in Experiment 3**.

Experiment	Signal color	SSD (*M[SD]*)
**Experiment 1**	White	313.00 ms (69.73)
	Red	323.63 ms (59.69)
	Green	314.72 ms (68.90)
**Experiment 2**	White	351.17 ms (64.44)
	Red	353.99 ms (46.63)
	Green	344.98 ms (54.40)
**Experiment 3**	Red	326.57 ms (68.30)
	Green	303.69 ms (70.40)

**Figure 2:**
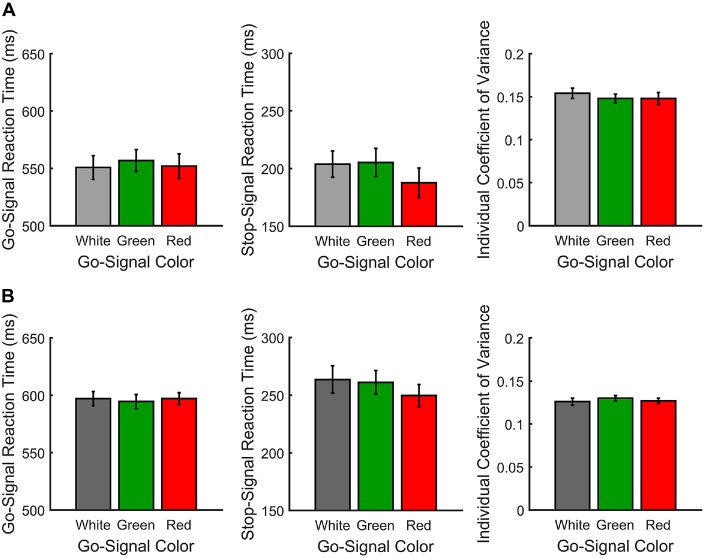
**Response measures for Experiments 1 (A)** and 2 **(B).** From left-to-right, go signal reaction time (RT; ms), individual coefficient of variance (ICOV) and stop signal reaction time (SSRT; ms) are shown as a function of go signal color.

Figure 2A shows the mean RTs, SSRTs and ICOVs for Experiment 1. In Experiment 1, irrelevant go-signal colors produced no significant difference for any of the metrics: mean RTs (*F*_(2,36)_ = 1.94, *p* = 0.159, $\eta_{\text{p}}^{\text{2}}$ = 0.097), mean ICOVs (*F*_(2,36)_ = 1.48, *p* = 0.240, $\eta_{\text{p}}^{\text{2}}$ = 0.076), mean SSD (*M[SD]* for white = 313.00 ms (69.73), red = 323.63 ms (59.69), green = 314.72 ms (68.90); *F*_(2,36)_ = 0.974, *p* = 0.387, *d*_RM_ = 0.051), SSRTs (*F*_(2,36)_ = 1.006, *p* = 0.376, $\eta_{\text{p}}^{\text{2}}$ = 0.053), or response accuracy (*M[SD]* for white = 92.68% (4.94), red = 92.53% (4.73), green = 94.16% (4.17); *F*_(2,36)_ = 2.032, *p* = 0.146, $\eta_{\text{p}}^{\text{2}}$ = 0.101). Those who were excluded from the final analysis had overall mean response accuracy rates below 50% (*M[SD]* for white = 45.32% (4.35), red = 43.24% (6.24), green = 44.89% (6.19)).

Paired *t*-tests for the planned comparisons revealed no significant differences between red and green go signals for any of the measures: mean RTs (*t*_(18)_ = 1.49, *p* = 0.155, *d*_RM_ = 0.358), mean ICOVs (*t*_(18)_ = −0.011, *p* = 0.991, *d*_RM_ < 0.003), mean SSD (*t*_(18)_ = 1.23, *p* = 0.236, *d*_RM_ < 0.293), SSRTs (*t*_(18)_ = −1.169, *p* = 0.258, *d*_RM_ < 0.27) or response accuracy (*t*_(18)_ = 1.946, *p* = 0.067, *d*_RM_ = 0.45). Overall, participants successfully countermanded responses on approximately 60% of stop trials (*M[SD]* for white = 60.74% (6.17), red = 59.37% (5.64), green = 60.42% (5.78)).

Figure 2B shows the mean RTs, SSRTs and ICOVs for Experiment 2. In Experiment 2, relevant go-signal colors also failed to produce a significant difference for any of the metrics: mean RTs (*F*_(2,42)_ = 0.173, *p* = 0.841, $\eta_{\text{p}}^{\text{2}}$ = 0.008), mean ICOVs (*F*_(2,42)_ = 0.395, *p* = 0.676, $\eta_{\text{p}}^{\text{2}}$ = 0.018), mean SSDs (*M[SD]* for white = 351.17 ms (64.44), red = 353.99 ms (46.63), green = 344.98 ms (54.40); *F*_(2,42)_ = 0.474, *p* = 0.626, $\eta_{\text{p}}^{\text{2}}$ = 0.022), SSRTs (*F*_(2,42)_ = 1.006, *p* = 0.376, $\eta_{\text{p}}^{\text{2}}$ = 0.053) or response accuracy (*M[SD]* for white = 80.59% (11.04), red = 82.64% (6.87), green = 82.18% (6.18); *F*_(2,42)_ = 0.696, *p* = 0.504, $\eta_{\text{p}}^{\text{2}}$ = 0.032). Note that response accuracy was slightly lower for Experiment 2 than in Experiment 1. This is likely due to the increase in possible responses from two arrow directions to three arrow colors. Those who were excluded from the final analysis had overall mean response accuracy rates below 33% (*M[SD]* for white = 29.68% (3.91), red = 31.12% (2.26), green = 29.96% (2.19)).

Paired *t*-tests for the planned comparisons revealed no significant differences between red and green go signals for any of the measures: mean RTs (*t*_(21)_ = 0.494, *p* = 0.626, *d*_RM_ = 0.107), mean ICOVs (*t*_(21)_ = 0.839, *p* = 0.411, *d*_RM_ = 0.179), mean SSDs (*t*_(21)_ = 1.22, *p* = 0.236, *d*_RM_ > 0.27), SSRTs (*t*_(21)_ = −0.575, *p* = 0.572, *d*_RM_ = 0.133) or response accuracy (*t*_(18)_ = −0.264, *p* = 0.795, *d*_RM_ < 0.06). Overall, participants successfully countermanded responses on approximately 60% of stop trials (*M[SD]* for white = 61.84% (6.08), red = 62.24% (6.55), green = 61.01% (6.19)).

As there was no significant effect of color regardless of color relevance, we combined the RT data from Experiments 1 and 2 in order to increase statistical power. The repeated measures ANOVA once again showed no significant effect of color on RT (*M[SD]* for white = 575.61 ms (43.74), red = 577.56 ms (39.61), green = 576.22 ms (42.65); *F*_(2,80)_ = 0.190, *p* = 0.827, $\eta_{\text{p}}^{\text{2}}$ = 0.005). The planned comparison between red and green also revealed no significant RT difference (*t*_(40)_ = 0.396, *p* = 0.694, *d*_RM_ < 0.063).

**Figure 3:**
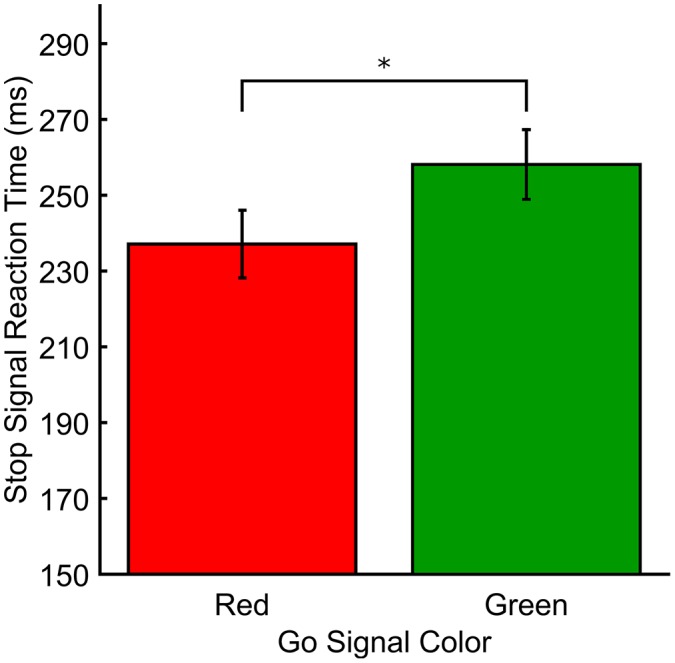
**Response measures for Experiment 3.** SSRT (ms) plotted as a function of stop signal color. *p < 0.05.

In Experiment 3, the stop-signal varied in color but as the go signal did not, there was no test of color on RTs (Mean RTs and mean ICOVs). Figure 3 shows the SSRTs for Experiment 3. The mean go signal RT was *M(SD)* = 556.89 ms (53.69). A paired *t*-test revealed that SSRTs were significantly faster for red (*M[SD]* = 237.12 ms [38.91]) relative to green (*M[SD]* = 258.20 ms [40.17]) stop-signals (*t*_(18)_ = −2.33, *p* = 0.031, *d*_RM_ = 0.331). There was also a significant difference in mean SSD (averaged across the entire experiment) between red (*M[SD]* = 326.57 ms [68.30]) and green (*M[SD]* = 303.69 ms [70.40]) stop signals in Experiment 3 (*t*_(18)_ = 3.036, *p* = 0.007, *d*_RM_ < 0.70). Effect sizes for paired *t*-tests (*d*_RM_) were calculated according to the method described in Morris and DeShon (2002). Mean go signal response accuracy for those included in the final analyses was *M(SD)* = 93.64% (4.65). Mean go signal response accuracy for those who were excluded from the final analyses was *M(SD)* = 46.91% (3.14). Overall, included participants successfully countermanded responses on approximately 55% of stop trials (*M[SD]* for red = 55.99% (6.44), green = 54.29% (5.32)).

## Discussion

In this study we were primarily interested in determining the relative effects of color on behavioral execution and response inhibition, as measured in the SST. Previous investigations revealed a color hierarchy of processing speed and strength with red stimuli producing faster and stronger processing than other colors (Lindsey et al., 2010; Tchernikov and Fallah, 2010; Pomerleau et al., 2014). According to the race model of executive function, the execution and cancelation of an action are independent processes which are in competition to reach threshold. Whichever process completes first determines the behavioral outcome (e.g., Logan and Cowan, 1984). Combining the color hierarchy and the race model, we found that color, whether task irrelevant (Experiment 1) or task relevant (Experiment 2), did not affect response execution. However, task relevant color did affect response inhibition as participants were 21 ms faster to countermand their response when the stop signal was red vs. green (Experiment 3).

This is a sizable difference in SSRTs between two signals that both indicate the response should be inhibited. This is likely due to the nature of the paradigm used in Experiment 3 and how it differs from other SSTs. Classically, the SST involves an auditory stop signal (e.g., Logan and Cowan, 1984), as we used in Experiments 1 and 2, separating the modalities of the go and stop signals. Other studies have used visual stop signals (Hanes and Schall, 1995; Hanes and Carpenter, 1999), which then compete for resources within the visual system. However, the visual stop signals are spatially separate from the go signal in a central-peripheral arrangement, which necessitates the involvement of spatial attention as well. Whether the stop signal is spatially separate in the visual domain, or in a separate modality, there is a need to monitor two sources of information. In our paradigm, a single object is both the go and stop signal. That object is processed and responded to, unless it undergoes an isoluminant color change, in which case the response is inhibited. As participants are focused on the color of a single object, this design strengthened the effects of the color hierarchy on task performance. We expect that if the color change occurred on a peripheral stimulus, the effects of the color hierarchy would be weaker, though the additional task demands could overwhelm the advantage for red completely.

Taken together, these findings provide for further understanding of executive functioning in general and the nature of the color hierarchy in particular. Faster search times, automatic target selection, greater pursuit gain, as well as stronger and faster propagation of ERPs for red stimuli relative to others can be explained by early biases in visual processing. The retina has a greater proportion of red cones than green or blue (e.g., Kuchenbecker et al., 2008). Thus there are more neural responses to red through the early visual system. Our findings suggest that this cannot be the only mechanism underlying the attention effects of color as this mechanism would result in facilitation for response execution as well as for response inhibition when comparing red to green. We propose that color is preferentially processed by neural circuits underlying response inhibition.

These results appear to contradict prior studies, as we did not find effects of color on response execution. Differences in task demands, however, explain this discrepancy. For example, in Lindsey et al. (2010) participants searched for red targets among distractors while in our study participants responded to a lone target. In other words, with competing stimuli, the attentional advantage for red results in more efficient search. This explanation is consistent with Pomerleau et al. (2014) who found that the N2PC waveform appears earlier and with greater amplitude for red compared to the other colors. The N2PC is an index of spatial filtering or surround suppression (Luck and Hillyard, 1994), of target feature enhancement at an attended location (Mazza et al., 2009), and as an index of the localization of a target prior to the deployment of attention (Tan and Wyble, 2015). Without competing stimuli, spatial filtering or surround suppression is not needed and so the speed of response execution would remain constant across colors. Conversely, spatial filtering or surround suppression cannot explain the effects on response inhibition determined by a color change. Therefore, the effects on response inhibition likely result from a mechanism not indexed by the N2PC.

However, it is possible that there may have been an effect on response execution that was masked by proactive response inhibition. Further investigations into the SST have shown that by having the potential for a stop trial randomly interleaved, go trials result in slower RTs (Verbruggen and Logan, 2009). This slowing of response execution is thought to be the result of preparatory activity in the response inhibition circuitry (Fassbender et al., 2006; Chikazoe et al., 2009), which increases the response thresholds (Verbruggen and Logan, 2009). It may be that if there were subtle effects of the color hierarchy on response execution, they may not have been strong enough to overcome the preparatory response inhibition.

### Putative Mechanism

The effect of color on response inhibition but not execution likely arises from the differential propagation of signals through separate neural pathways. In the horse-race model, Logan and Cowan (1984) posited that execution and inhibition are separate processes which race to reach some threshold. Whichever process reaches threshold first wins the race and determines the behavioral outcome. Recent neurophysiological work has provided physical evidence for functionally distinct but interacting subcortical (i.e., basal ganglia) pathways for starting and stopping actions, termed the direct, indirect and hyperdirect pathways. The direct pathway facilitates behavioral execution by inhibiting the effects of the substantia nigra pars reticulata (SNr) on the thalamus. This allows for voluntary movements to be released and executed through thalamic excitation of the motor cortex (e.g., Albin et al., 1989). This pathway was not affected by task-relevant or irrelevant color (Experiments 1 and 2). The indirect pathway involves inhibition of the globus pallidus which leads to the SNr increasing inhibition of the motor output centers of the thalamus. This has the effect of inhibiting motor excitation and thus it facilitates behavioral inhibition. As the same basal ganglia are involved as for response execution, it is unlikely that color could play a role in this pathway. However, a third “hyperdirect pathway” has also been proposed (e.g., Nambu et al., 2002). In this pathway, excitatory prefrontal cortical input is fed directly into the subthalamic nucleus (STN) which brings about the excitation of the SNr and globus pallidus. This has the double effect of modulating cortical input to the basal ganglia while also inhibiting output from the thalamus to the motor cortex. The hyperdirect pathway is named as such because it includes a more direct pathway from the cortex to the STN but also because the interneuron cascade of events occurs more quickly. The fast action of the hyper-direct pathway might preserve the advantage for red, therefore resulting in better inhibitory control, while the direct pathway may not, resulting in no differential effect on behavioral execution time.

### Functional Advantage

While the color hierarchy was initially elucidated using red, green, yellow and blue stimuli, these experiments focused on red and green as these colors are often used in the execution or inhibition of an action (e.g., red meaning stop and green meaning go). The color red, in particular, may have been developed for use as a stop-signal because it is often associated with danger, such as poisonous berries and frogs, the color of blood, or changes in skin tone when someone is angry. In these situations, it would be advantageous to inhibit a current behavior in order to perform an alternate action (i.e., the hierarchy could be inherent) Alternatively, as red and green have many modern associations (e.g., traffic lights, elevator panels, user interfaces), the effects of color on response inhibition may have arisen from experience (i.e., the hierarchy may be learned). In fact, the evolutionary and the experiential effects may both be in effect. This is important because there is evidence to suggest that response inhibition may be facilitated when subjects are repeatedly exposed to a particular stimulus stop association (e.g., Verbruggen and Logan, 2008). Though it should be noted that if training were solely responsible for the color effects, we should have found a RT advantage for green stimuli as they are typically used to bring about behavioral execution (e.g., traffic light). Further experiments are necessary to elucidate the underlying neural circuitry that we are proposing.

It is important to note that our results and the results of previous color hierarchy studies add to a host of research showing that the color red in particular has a special modulatory effect on behavior and cognition. For example, the color red degrades performance in achievement contexts (e.g., tests; Elliot et al., 2007; Maier et al., 2008), red cues can interact with the emotional valence of stimuli to modulate responses to emotional stimuli (Kuniecki et al., 2015), and females who wear red clothing are rated as being more attractive and as having more sexual intent by heterosexual males, suggesting that red may act as a sexual cue (e.g., Guéguen, 2012; Elliot et al., 2013).

## Conclusion

We have shown here for the first time that the color hierarchy affects higher level motor decision making circuits. Interestingly, there is no differential effect of go-signal color on response execution times. For response countermanding, however, SSRTs show that red signals allow for participant to countermand response execution an average of 25 ms faster than green signals. This provides further evidence both for an automatic color hierarchy and for the dissociation between execution and inhibition networks, where color is preferentially processed by circuits underlying response inhibition. Importantly, our findings also show that the color hierarchy is not the result of biases in early visual processing but, rather, that it is likely due to higher level attentional networks.

## Author Contributions

SB wrote the manuscript, collected and analyzed the data, and aided in the programming of the experiments. AF-R collected data and provided insight into the psychological aspects of color perception. MF devised the experiments, aided in the programming of the experiments and provided editorial support for the manuscript.

## Funding

This work was funded by an Natural Sciences and Engineering Research Council of Canada (NSERC) Discovery Grant to MF and a MITACS Globalink Summer Research Internship to AF-R.

## Conflict of Interest Statement

The authors declare that the research was conducted in the absence of any commercial or financial relationships that could be construed as a potential conflict of interest.
